# Complete Mitochondrial Genome of Three *Bactrocera* Fruit Flies of Subgenus *Bactrocera* (Diptera: Tephritidae) and Their Phylogenetic Implications

**DOI:** 10.1371/journal.pone.0148201

**Published:** 2016-02-03

**Authors:** Hoi-Sen Yong, Sze-Looi Song, Phaik-Eem Lim, Praphathip Eamsobhana, I. Wayan Suana

**Affiliations:** 1 Institute of Biological Sciences, University of Malaya, Kuala Lumpur, Malaysia; 2 Chancellory High Impact Research, University of Malaya, Kuala Lumpur, Malaysia; 3 Institute of Ocean and Earth Sciences, University of Malaya, Kuala Lumpur, Malaysia; 4 Department of Parasitology, Faculty of Medicine Siriraj Hospital, Mahidol University, Bangkok, Thailand; 5 Faculty of Science and Mathematics, Mataram University, Mataram, Indonesia; Sichuan University, CHINA

## Abstract

*Bactrocera latifrons* is a serious pest of solanaceous fruits and *Bactrocera umbrosa* is a pest of Artocarpus fruits, while *Bactrocera melastomatos* infests the fruit of Melastomataceae. They are members of the subgenus *Bactrocera*. We report here the complete mitochondrial genome of these fruit flies determined by next-generation sequencing and their phylogeny with other taxa of the subgenus *Bactrocera*. The whole mitogenomes of these three species possessed 37 genes namely, 13 protein-coding genes (PCGs), 2 rRNA and 22 tRNA genes. The mitogenome of *B*. *latifrons* (15,977 bp) was longer than those of *B*. *melastomatos* (15,954 bp) and *B*. *umbrosa* (15,898 bp). This difference can be attributed to the size of the intergenic spacers (283 bp in *B*. *latifrons*, 261 bp in *B*. *melastomatos*, and 211 bp in *B*. *umbrosa*). Most of the PCGs in the three species have an identical start codon, except for *atp8* (adenosine triphosphate synthase protein 8), which had an ATG instead of GTG in *B*. *umbrosa*, whilst the *nad3* (NADH dehydrogenase subunit 3) and *nad6* (NADH dehydrogenase subunit 6) genes were characterized by an ATC instead of ATT in *B*. *melastomatos*. The three species had identical stop codon for the respective PCGs. In *B*. *latifrons* and *B*. *melastomatos*, the TΨC (thymidine-pseudouridine-cytidine)-loop was absent in *trnF* (phenylalanine) and DHU (dihydrouracil)-loop was absent in *trnS1* (serine S1). In *B*. *umbrosa*, *trnN* (asparagine), *trnC* (cysteine) and *trnF* lacked the TψC-loop, while *trnS1* lacked the DHU-stem. Molecular phylogeny based on 13 PCGs was in general concordant with 15 mitochondrial genes (13 PCGs and 2 rRNA genes), with *B*. *latifrons* and *B*. *umbrosa* forming a sister group basal to the other species of the subgenus *Bactrocera* which was monophyletic. The whole mitogenomes will serve as a useful dataset for studying the genetics, systematics and phylogenetic relationships of the many species of *Bactrocera* genus in particular, and tephritid fruit flies in general.

## Introduction

Fruit flies in the genus *Bactrocera* are potentially destructive pests of commercial fruits and vegetables [1]. Seventy-three species have been documented as economically important in the Pacific Region [2]. Seven out of the nine species are rated as the most serious pests (Category A) [2], these being members of the subgenus *Bactrocera*; the other two less harmful species are *B*. (*Daculus*) *oleae* (Gmelin) and *B*. (*Zeugodacus*) *cucurbitae* (Coquillett). *B*. *latifrons* (Hendel) is one of the seven pest species belonging to the subgenus *Bactrocera*; the other members are *B*. (*B*.) *carambolae* Drew and Hancock, *B*. (*B*.) *correcta* (Bezzi), *B*. (*B*.) *dorsalis* (Hendel), *B*. (*B*.) *neohumeralis* (Hardy), *B*. (*B*.) *tryoni* (Froggatt), and *B*. (*B*.) *zonata* (Saunders). *Bactrocera latifrons* fruit hosts are mainly Solanaceae and Cucurbitaceae although 59 plant species from 14 plant families have been documented [3]. This pest has a broad geographical range occurring in Pakistan, India, Sri Lanka, Myanmar, China, Taiwan, Thailand, Laos, Vietnam, Malaysia, Singapore, Brunei, and Indonesia, and has been introduced into Hawaii, Okinawa, Tanzania, and Kenya [2–6].

A less serious pest species of the subgenus *Bactrocera* is *B*. *umbrosa* (Fabricius)–one of 16 species in Category C consisting of relatively minor oligophagous or specialist fruit or cucurbit pests [2]. It infests Artocarpus fruits and is widespread from southern Thailand through New Guinea to New Caledonia [2]. Another species, *B*. *melastomatos* Drew & Hancock of the subgenus *Bactrocera* is not known to damage commercial crop plants but infests the fruit of Melastomataceae [7]. It has been documented in India (Andaman Island), Thailand, Peninsular Malaysia, Singapore, and Indonesia (Java, Kalimantan, Sumatra) [7].

There are few reports on the molecular phylogeny of *B*. *latifrons*, *B*. *melastomatos* and *B*. *umbrosa*. Based on 16S rRNA and cytochrome oxidase I nucleotide sequences, *B*. *latifrons* shows close affinity to *B*. *umbrosa* and is most basal to subgenus *Bactrocera* [8]. In another study based on cytochorome oxidase I, *B*. *umbrosa* forms a sister group with *B*. *facialis* while *B*. *latifrons* is basal to subgenus *Bactrocera* [9]. Based on 17 enzyme loci profile using starch-gel electrophoresis, *B*. *melastomatos* is distinct from the lineage of *B*. *dorsalis* and *B*. *carambolae* [10].

To date, the complete mitochondrial genomes (mitogenomes) of six species of the subgenus *Bactrocera*–*B*. *arecae*, *B*. *carambolae*, *B*. *correcta*, *B*. *dorsalis* (incuding the conspecific taxa *B*. *papayae* and *B*. *philippinensis*), *B*. *tryoni*, and *B*. *zonata*–are available in GenBank. We report here the mitogenome of three additional species of the subgenus (*B*. *latifrons*, *B*. *melastomatos*, and *B*. *umbrosa*) determined by next-generation sequencing and their phylogenetic relationships with other taxa of the subgenus *Bactrocera*.

## Materials and Methods

### Ethics statement

*B*. *latifrons*, *B*. *melastomatos* and *B*. *umbrosa* are insect pests. They are not endangered or protected by law. No permits are required to study these fruit flies.

### Specimen Collection

Fruit flies of *B*. *latifrons* were hatched from infested chilli fruit (*Capsicum annuum*) collected in University of Malaya campus [11]. Male fruit flies of *B*. *melastomatos* were collected by means of Cue lure [12] and *B*. *umbrosa* by means of methyl eugenol in University of Malaya campus. The specimens were preserved in 95% absolute ethanol and stored in -20°C freezer until use.

### Mitochondria isolation and DNA extraction

A small piece of the alcohol-preserved tissue of each *Bactrocera* species was pressed onto a C-fold paper towel to remove excess ethanol before homogenisation. The mitochondria were isolated by standard differential centrifugation method [13] and the mtDNA was extracted using Mitochondrial DNA Isolation Kit (Abnova, Taipei, Taiwan) following the manufacturer’s instructions with minor modification. The mtDNA was eluted using 30 ul elution buffer instead of Tris-EDTA (TE) buffer to avoid interference of Ethylenediaminetetraacetic acid (EDTA) with the enzyme such as transposases.

### Sample and library preparation

The purified mtDNA was quantified using Qubit dsDNA High Sensitivity Assay Kit (Life Technologies, USA) and normalized to a final concentration of 50 ng (20 ul of mtDNA at 2.5 ng/ul). Library was prepared using Nextera DNA Sample Preparation Kit (Illumina, USA) following the manufacturer’s protocols. Size estimation of the library was performed on a 2100 Bioanalyzer using High Sensitivity DNA Analysis Kit (Agilent Technologies). The library was quantified with Qubit 2.0 Fluorometer (Life Technologies, USA).

### Genome Sequencing

The library was normalized to 12 picomolar and sequenced using the NextSeq 500 Dekstop Sequencer (2 × 150 bp paired-end reads) (Illumina, USA).

### Sequence and genome analysis

Raw sequence reads were extracted from the Illumina NextSeq 500 system in FASTQ format. The quality of sequences was evaluated using the FastQC software [14]. All ambiguous nucleotides and reads with an average quality value lower than Q20 were excluded from further analysis. The trimmed sequences were mapped against three reference mitogenomes namely, *Bactrocera dorsalis* (NC_008748), *B*. *tryoni* (NC_014611) and *B*. *zonata* (NC_027725) using the CLC Genomic Workbench version 8.0.1 (Qiagen, Germany) with mapping parameters of length fraction = 0.6 and similarity fraction = 0.7. The mapped sequences were then subjected to de novo assembly. Contigs greater than 15 kbp were subjected to BLAST [15] alignment against the nucleotide database at National Center for Biotechnology Information (NCBI). Contigs with hits to mitochondrial genes or genomes were identified and extracted using the CLC Genomic Workbench interface.

### Mitogenome identification, annotation and visualization

A single contig which blasted as mitochondrial sequence was manually examined for repeats at the beginning and end of the sequence to establish a circular mtDNA. It was then annotated with MITOS [16] followed by manual validation of the coding regions. Open reading frames (ORFs) were predicted using the NCBI ORF Finder (http://www.ncbi.nlm.nih.gov/gorf/gorf.html). The sequin file generated from MITOS was edited and submitted to NCBI according to ORF Finder result and can be accessed at NCBI GenBank using the accession numbers: *Bactrocera latifrons* KT881556; *Bactrocera melastomatos* KT881557; and *Bactrocera umbrosa* KT881558. The circular mitogenome was visualized with Blast Ring Image Generator (BRIG) [17].

### Mitogenomes from GenBank

The mitogenomes of Tephritidae available from GenBank (*Bactrocera dorsalis* NC_008748, NC_009790, NC_009771; *B*. *carambolae* NC_009772; *B*. *arecae* KR233259; *B*. *correcta* NC_018787; *B*. *tryoni* NC_014611; *B*. *zonata* NC_027725; *B*. *oleae* NC_005333; *B*. *minax* NC_014402; *B*. *cucurbitae* NC_016056; *B*. *scutellata* NC_027254; *B*. *tau* NC_027290; *B*. *caudata* Malaysia KT625491; *B*. *caudata* Indonesia KT625492; *Ceratitis capitata* NC_000857; *Procecidochares utilis* NC_020463) were used for phylogenetic comparison. Species of *Drosophila* (*D*. *incompta* NC_025936; *D*. *melanogaster* NC_024511; *D*. *yakuba* NC_001322) were used as outgroup taxa.

### Phylogenetic analysis

The total length of the aligned sequences of each mitogenome comprised of 13 protein-coding genes (PCGs), 2 rRNA genes and 15 mt-genes (13 PCGs, 2 rRNA genes). This data as well as the selected models used for maximum likelihood (ML) and Bayesian Inference (BI) analyses are summarized in Table 1.

**Table 1:** Information of the aligned sequences of 13 protein-coding genes (PCGs), 2 rRNA genes, and 13 PCGs + 2 rRNA genes of *Bactrocera latifrons*, *B*. *melastomatos*, *B*. *umbrosa* and related taxa. AIC, Akaike Information Criterion; BIC, Bayesian Information Criterion.

Data set	No. taxa	Total length (bp)	Model selected based on AIC	Model selected based on BIC
15 mt-genes	23	13427	GTR+Gamma	SYM+Gamma
13 PCGs	23	11217	GTR+Gamma	SYM+Gamma
2 rRNA genes	23	2210	GTR+Gamma	SYM+Gamma

The 13 PCG sequences were separately aligned using ClustalX v.1.81 program [18] and were subsequently edited and trimmed using BioEdit v.7.0.5.3 [19]. The sequences of the large-(*rrnL*) and small-(*rrnS*) subunit genes were aligned using MAFFT v.7 [20] (The aligned sequences can be given upon request). Kakusan v.3 [21] was used to determine the best-fit nucleotide substitution models for maximum likelihood (ML) and Bayesian (BI) analyses based on the corrected Akaike Information Criterion [22] and the Bayesian Information Criterion [23], respectively.

Phylograms of 13 concatenated PCGs, 2 rRNA genes and 15 mt-genes were constructed using TreeFinder [24]. Bootstrap values (BP) were generated via 1,000 ML bootstrap replicates. Bayesian analyses were conducted using the Markov chain Monte Carlo (MCMC) method via Mr. Bayes v.3.1.2 [25], with two independent runs of 2×10^6^ generations with four chains, and with trees sampled every 200^th^ generation. Likelihood values for all post-analysis trees and parameters were evaluated for convergence along with burn-in (a specified number of samples from the beginning of the chain to be discarded) using the “sump” command in MrBayes and the computer program Tracer v.1.5 (http://tree.bio.ed.ac.uk/software/tracer/). The first 200 trees from each run were discarded as burn-in (where the likelihood values were stabilized prior to the burn-in), and the remaining trees were used for the construction of a 50% majority-rule consensus tree. Phylogenetic trees were viewed and edited by FigTree v.1.4 [26]. To assess the level of variation, uncorrected pairwise (p) genetic distances were estimated using PAUP* 4.0b10 software [27].

## Results

### Mitogenome analysis and features

The sequencing reads, GC content and base composition of *Bactrocera* mitogenomes produced by next-generation sequencing on Illumina NextSeq 500 Desktop Sequencer are summarized in Table 2.

**Table 2:** Number of reads, GC content and base composition of *Bactrocera* mitogenomes produced by next-generation sequencing.

Taxon	Raw reads	Final reads*	GC content (%)	Base composition (%)			
				A	T	G	C
*B*. *latifrons*	37,672,394	28,041,251	28.9	38.7	32.4	10.6	18.3
*B*. *melastomatos*	35,958,110	28,357,462	26.2	39.6	34.2	9.8	16.4
*B*. *umbrosa*	39,220,792	28,401,168	29.5	38.2	32.3	11.2	18.3

* after removal of low quality sequence (< Q20) and sequences shorter than 50 nucleotides.

**Fig 1:**
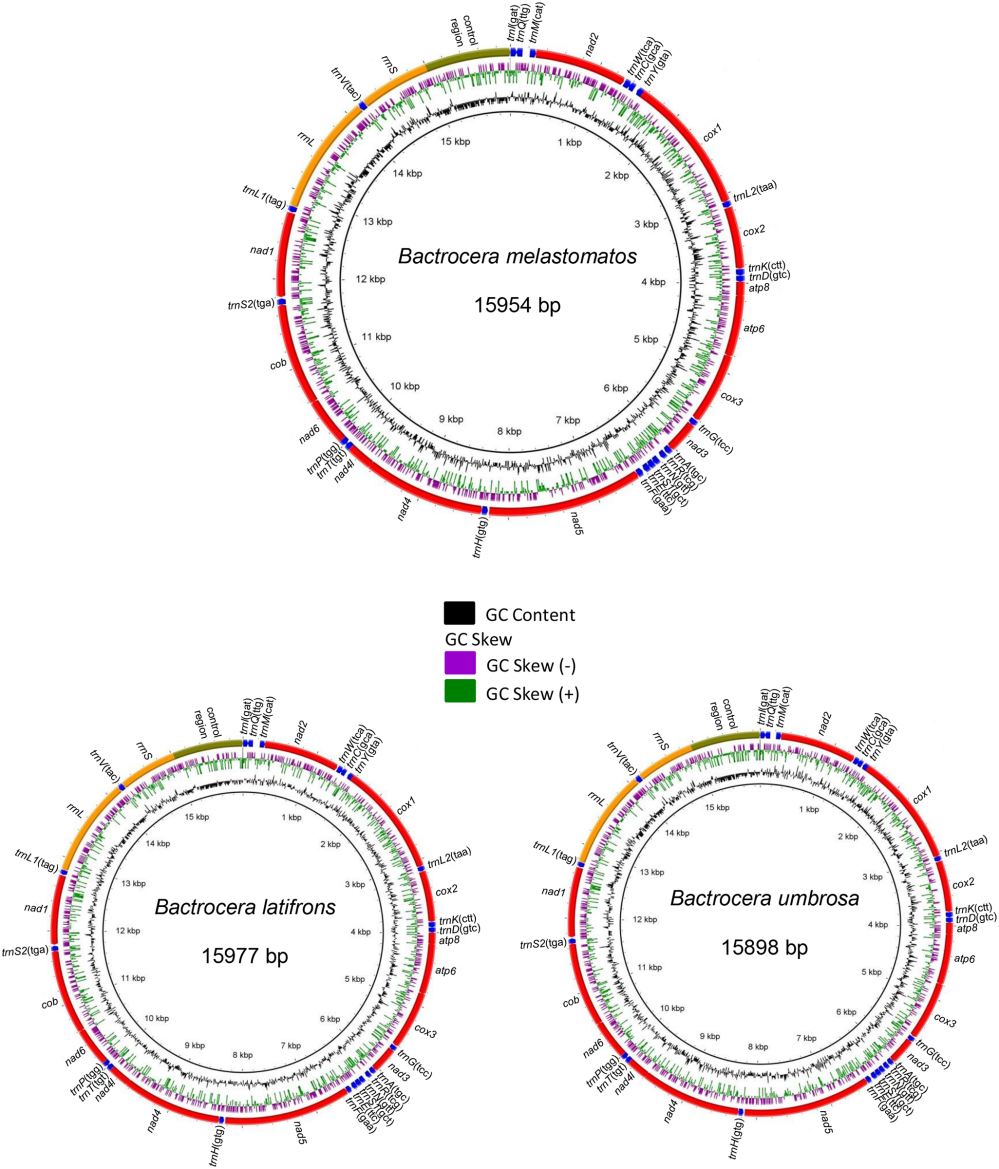
Complete mitogenomes of *Bactrocera latifrons*, *B*. *melastomatos* and *B*. *umbrosa* with BRIG visualization showing the protein-coding genes, rRNAs and tRNAs. GC skew is shown on the outer surface of the ring whereas GC content is shown on the inner surface. The anticodon of each tRNAs is shown in bracket.

The mitogenomes of *B*. *latifrons*, *B*. *melastomatos* and *B*. *umbrosa* had similar gene order and contained 37 genes (13 protein-coding genes—PCGs, 2 rRNA genes, and 22 tRNA genes) and a non-coding region (A + T-rich control region) (Fig 1, S1–S3 Tables). Control region was flanked by *rrnS* and *trnI* genes respectively, with 953 bp in *B*. *latifrons* and *B*. *melastomatos*, and 944 bp in *B*. *umbrosa*. A long polyT-stretch of 23 bp in *B*. *latifrons*, 20 bp in *B*. *melastomatos*, and 24 bp in *B*. *umbrosa* was observed.

There were 15 intergenic regions with spacing sequence totalling 283 bp in *B*. *latifrons*, 261 bp in *B*. *melastomatos*, and 211 bp in *B*. *umbrosa*. The region between *trnQ* and *trnM* genes was separated by 94 bp in *B*. *latifrons*, 82 bp in *B*. *melastomatos*, and 79 bp in *B*. *umbrosa*. Sequences with 39, 43 and 94 bases in *B*. *latifrons*, 35, 39 and 82 bases in *B*. *melastomatos*, and 79 bases in *B*. *umbrosa* had clear stem-loop structures. All the three species had overlaps in seven regions totalling 29 bp.

The three species shared an identical start codon for most of the PCGs, except ATG (instead of GTG) for *atp8* in *B*. *umbrosa*, and ATC (instead of ATT) for *nad3* and *nad6* in *B*. *melastomatos* (S4 Table). Of the start codons common to the three species, the commonest was ATG (in 6 PCGs–*cox2*, *atp6*, *cox3*, *nad4*, *nad4l*, *cob*), followed by two ATT (*nad2*, *nad5*) and one each for ATA (*nad1*) and TCG (*cox1*). The three species had an identical stop codon for the respective PCGs (S4 Table). Seven PCGs has a TAA stop codon (*nad2*, *cox2*, *atp8*, *atp6*, *cox3*, *nad4l*, *nad6*), one had TAG (*nad4*), and five had truncated stop codon (1 TA–*cox1*; 4 T–*nad3*, *nad5*, *cob*, *nad1*).

The nucleotide compositions of the mitochondrial whole genome, protein-coding genes, rRNA genes and control region of *B*. *latifrons*, *B*. *melastomatos* and *B*. *umbrosa* are summarized in S5–S7 Tables. All three species were A+T rich as expected for mitochondrial genomes. The A + T content for PCGs was lowest in *cox1* (61.8% for *B*. *latifrons*, 65.0% for *B*. *melastomatos*, and 60.5% for *B*. *umbrosa*) and highest in *nad4l* (76.4% for *B*. *latifrons* and 74.4% for *B*. *umbrosa*) and *nad6* (78.7% for *B*. *melastomatos*). The A + T content of the non-coding control region was 86.8% for *B*. *latifrons*, 89.0% for *B*. *melastomatos* and 86.2% for *B*. *umbrosa*. For the two ribosomal operons, *rrnL* had a higher A + T content than *rrnS* (78.9% vs 74.4% for *B*. *latifrons*, 80.2% vs 74.6% for *B*. *melastomatos*, and 79.0% vs 73.6% for *B*. *umbrosa*). The GC skew content which included the whole genome, PCGs, rRNA genes and control region in the three species were negative indicating a bias toward the use of Cs over Gs. Although the AT skewness value was positive for the whole genome, rRNA genes and control region, it was variable in the individual PCGs.

As in other insects, the mitogenomes of *B*. *latifrons*, *B*. *melastomatos* and *B*. *umbrosa* had three main tRNA clusters which are characteristically depicted in Fig 1. These include: (1) I-Q-M; (2) W-C-Y; and (3) A-R-N-S1-E-F (Fig 1). The cloverleaf structure for the respective tRNAs was similar in *B*. *latifrons* and *B*. *melastomatos*. The TΨC-loop was absent in *trnF* while *trnS1* lacked the DHU-loop (S1 and S2 Figs). In *B*. *umbrosa*, *trnN*, *trnC* and *trnF* lacked the TψC-loop, while *trnS1* lacked DHU-stem (S3 Fig).

### Phylogenetic relationships and genetic divergence

**Fig 2:**
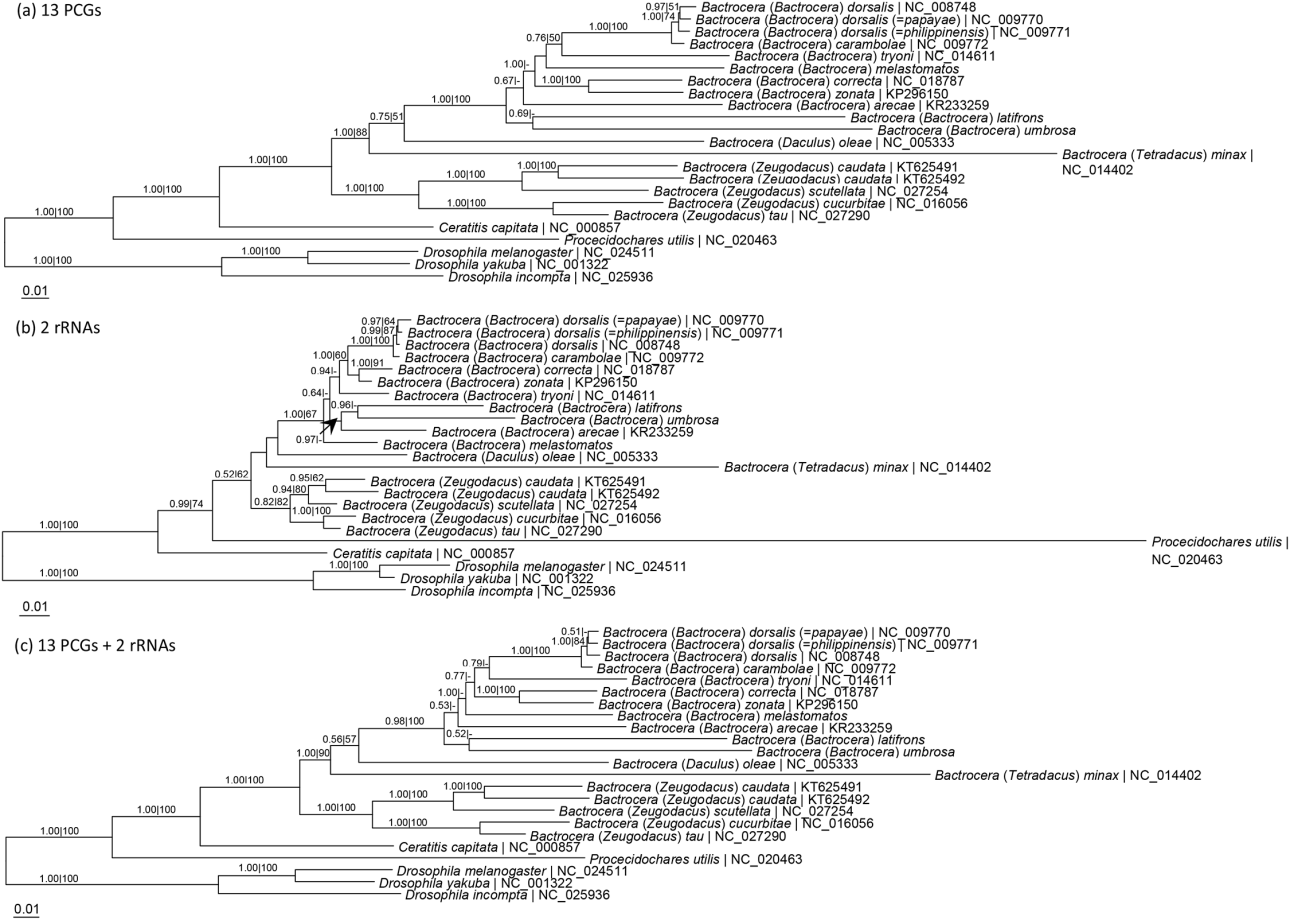
Maximum likelihood tree based on (a) 13 protein-coding genes, (b) 2 rRNA genes, and (c) 13 PCGs and 2 rRNA genes of the whole mitogenomes of *Bactrocera* taxa of the subgenus *Bactrocera* and other Tephritid fruit flies with Drosophilidae as outgroup. Numeric values at the nodes are Bayesian posterior probabilities/ML bootstrap. Figures 3 and 4 in Yong et al. (2015) [10] were interposed.

The molecular phylogeny of *B*. *latifrons*, *B*. *melastomatos* and *B*. *umbrosa* in relation to other *Bactrocera* taxa of the subgenus *Bactrocera* and other Tephritidae are shown in Fig 2. The phylogram based on 13 PCGs was in general congruent with that based on 15 mt-genes, except for the position of *B*. *melastomatos*. The subgenus *Bactrocera* was monophyletic, forming a distinct clade from the other *Bactrocera* taxa in which the subgenus *Zeugodacus* was monophyletic. Of the three species in the present study, *B*. *latifrons* and *B*. *umbrosa* formed a sister group and were basal to the other taxa of the subgenus *Bactrocera*.

The genetic diversity of *B*. *latifrons*, *B*. *melastomatos*, *B*. *umbrosa* and related taxa of the subgenus *Bactrocera* based on (a) 13 PCGs, (b) 2 rRNA genes, and (c) 13 PCGs + 2 rRNAs genes is summarized in Table 3.

**Table 3:** Percentage of uncorrected pairwise (p) genetic distance between different pairs of *Bactrocera* taxa of the subgenus *Bactrocera* based on (a) 13 protein-coding genes (PCGs), (b) 2 rRNA genes, and (c) 13 PCGs + 2 rRNA genes.

Species pair	(a) 13 PCGs	(b) 2 rRNAs	(c) 13 PCGs + 2 rRNAs
*B*. *latifrons* KT881556 vs *B*. *umbrosa* KT881558	15.4	9.0	14.2
*B*. *latifrons* KT881556 vs *B*. *melastomatos* KT881557	13.3	6.1	12.2
*B*. *latifrons* KT881556 vs *B*. *carambolae* NC_009772	13.0	5.7	11.8
*B*. *latifrons* KT881556 vs *B*. *arecae* KR233259	13.2	6.3	12.1
*B*. *latifrons* KT881556 vs *B*. *dorsalis* NC_008748	13.0	5.8	11.8
*B*. *melastomatos* KT881557 vs *B*. *umbrosa* KT881558	13.6	6.5	12.5
*B*. *melastomatos* KT881557 vs *B*. *arecae* KR233259	10.6	4.7	9.6
*B*. *melastomatos* KT881557 vs *B*. *carambolae* NC_009772	9.2	3.9	8.4
*B*. *melastomatos* KT881557 vs *B*. *dorsalis* NC_008748	9.1	4.0	8.3
*B*. *carambolae* NC_009772 vs *B*. *dorsalis* NC_008748	1.4	0.4	1.2
*B*. *arecae* KR233259 vs *B*. *tryoni* NC_014611	10.3	4.6	9.4
*B*. *correcta* NC_018787 vs *B*. *zonata* KP296150	6.3	1.6	5.5

## Discussion

Mitochondrial genomes of insects are extensively studied with particular reference to their phylogenetic and evolutionary studies [28]. The use of heterogeneous CAT and CAT 1 GTR models indicates that the complete nucleotide sequences (PCG and PCGRNA) of mitogenome are suitable for resolving higher-level phylogeny of Paraneopteran insects [29]. To date there are complete mitogenomes for six species of the subgenus *Bactrocera* namely, *B*. *dorsalis*, *B*. *carambolae*, *B*. *arecae*, *B*. *correcta*, *B*. *tryoni*, and *B*. *zonata*. The present study has added three more species to this list.

The mitogenome size of *B*. *umbrosa* (15,898 bp) is smaller than those of *B*. *latifrons* (15,977 bp) and *B*. *melastomatos* (15,954 bp) (S1–S3 Tables). This is due mainly to the size of the intergenic spacers– 211 bp in *B*. *umbrosa*, 261 bp in *B*. *melastomatos* and 283 bp in *B*. *latifrons*. Among the mitogenomes of the subgenus *Bactrocera* available in GenBank, *B*. *dorsalis* (including the conspecific *B*. *papayae* and *B*. *philippinensis*) and *B*. *carambolae* have a mitogenome size of 15,915 bp, *B*. *tryoni* 15,925 bp, *B*. *zonata* 15,935 bp, and *B*. *correcta* 15,936 bp respectively.

The start and stop codons for the respective PCGs in the nine *Bactrocera* taxa of the subgenus are not invariant (S4 Table). They are identical in seven PCGs–*nad2*, *cox1*, *cox2*, *cox3*, *nad4*, *nad4l*, and *nad1* (S4 Table). In this study, *B*. *umbrosa* differs from the other species in the possession of ATG (instead of GTG) start codon for *atp8*. *B*. *melastomatos* differs from the other species in having ATC (instead of ATT) start codon for *nad3* and *nad6*.

Seven PCGs (*cox1*, *atp6*, *nad3*, *nad5*, *nad6*, *cob*, *nad1*) have incomplete stop codons in some members of the nine *Bactrocera* taxa of the subgenus *Bactrocera* (S4 Table); only TA for *cox1* and T for *nad1* are present in all the nine taxa. The incomplete stop codons (T and TA) can be converted to TAA by post-translational polyadenylation [30].

Among the tRNAs, *trnF* lacks the TΨC-loop in all the nine *Bactracera* taxa of the subgenus *Bactrocera* (Table 4). Two other tRNAs also lack the TΨC-loop–*trnN* in *B*. *umbrosa*, *B*. *arecae*, *B*. *dorsalis*, *B*. *carambolae* and *B*. *tryoni*; and *trnC* in *B*. *umbrosa*, *B*. *dorsalis*, *B*. *carambolae* and *B*. *tryoni*. *trnS1* has aberrant cloverleaf structure for DHU arm, lacking DHU-stem in *B*. *umbrosa* and DHU-loop in eight of the nine taxa of the subgenus *Bactrocera* (Table 4). Deviant tRNA secondary structures are particularly frequent in Arthropoda [31]. The TΨC-loop and DHU-loop of tRNA act as special recognition site during protein biosynthesis or translation [32–34]. It has been reported that misacylation of tRNA can affect the survivability of an organism [34].

**Table 4:** Absence of TΨC-loop, DHU-loop and DHU-stem in the transfer RNAs of *Bactrocera* taxa of the subgenus *Bactrocera*.

Taxon	*trnN* TΨC-loop absent	*trnC* TΨC-loop absent	*trnF* TΨC-loop absent	*trnS1* DHU-loop absent	*trnS1* DHU-stem absent
***B*. *latifrons***			●	●	
***B*. *melastomatos***			●	●	
***B*. *umbrosa***	●	●	●		●
***B*. *arecae***	●		●	●	
***B*. *correcta***			●	●	
***B*. *dorsalis***	●	●	●	●	
***B*. *carambolae***	●	●	●	●	
***B*. *tryoni***	●	●	●	●	
***B*. *zonata***			●	●	

● indicates absence.

Studies on molecular phylogeny of *Bactrocera* fruit flies have been based mainly on mitochondrial and nuclear genes, e.g. the phylogenetic relationships among (1) 24 *Bactrocera* species based on *rrnL*, *cox2*, *trnK* and *trnD* genes [35], (2) 125 Dacini species based on *rrnL*, *cox1*, *cox2* and “white-eye” genes [36], (3) 47 *Bactrocera* species based on *cox1* gene sequences [37], and (4) 56 *Bactrocera* taxa using *cox1* and *rrnL* gene fragments [38].

Molecular studies have revealed considerable variation in genetic diversity among closely related taxa of *Bactrocera* fruit flies. A recent study based on six loci (*cox1*, *nad4-*3′, CAD, *period*, ITS1, ITS2) indicates that *B*. *dorsalis s*.*s*., *B*. *papayae* and *B*. *philippinensis* are the same biological species [39]. Another taxon *B*. *invadens* has also been synonymized with *B*. *dorsalis* [40]. Based on analysis of 13 PCGs, the uncorrected genetic ‘p’ distance is 1.06 between *B*. *dorsalis* and *B*. *dorsalis* (= *papayae*) and 1.11 between *B*. *dorsalis* and *B*. *dorsalis* (= *philippinensis*) [10]. Analyses of the *cox1*, *cox2*, *rrnL* and concatenated *cox1*+*cox2*+*rrnL* and *cox1*+*cox2*+*rrnL*+28S+ITS-2 nucleotide sequences reveal that *B*. *caudata* from the northern hemisphere (Peninsular Malaysia, East Malaysia, Thailand) and southern hemisphere (Indonesia: Java, Bali and Lombok) are genetically distinct, with uncorrected ‘p’ distance of 4.46–4.94% for the concatenated *cox1*+*cox2*+*rrnL* nucleotide sequences which is several folds higher than the ‘p’ distance for the taxa in the northern hemisphere (‘p’ = 0.00–0.77%) and the southern hemisphere (‘p’ = 0.00%) [12].

Two recent studies on the mitogenomes of *Bactrocera* fruit flies of the subgenus *Bactrocera* have reported the sister lineage of *B*. *correcta* and *B*. *zonata* [41] and that of *B*. *arecae* and *B*. *tryoni* [10], in addition to the sister lineage of *B*. *dorsalis* and *B*. *carambolae*. The present results of *B*. *melastomatos* being distinct from the lineage of *B*. *dorsalis* and *B*. *carambolae* agree with earlier finding based on 17 enzyme loci profile using starch-gel electrophoresis [9].

In the present study, the subgenus *Bactrocera* is monophyletic (Fig 2). Of the other subgenera, *B*. (*Daculus*) *oleae* and *B*. (*Tetradacus*) *minax* form a clade with subgenus *Bactrocera*, while the subgenus *Zeugodacus* forms a distinct clade (Fig 2).

Based on 13 PCGs and 15 mt-genes, *B*. *latifrons* and *B*. *umbrosa* form a sister group basal to the other members of the subgenus *Bactrocera* (Fig 2). This finding concurs with that based on *rrnL* and *cox1* sequences [8]. However, it differs from that based on *cox1* gene which reveals *B*. *latifrons* is most basal to the subgenus *Bactrocera* but does not form a lineage with *B*. *umbrosa* [38]. The species tree differs from the finding based on *cox1*, *rrnL*, *trnP*, *nad6* and *period* genes in which *B*. *latifrons* and *B*. *umbrosa* do not form a sister lineage [42]. With the inclusion of *B*. *latifrons*, the present finding helps to resolve the inference of *B*. *umbrosa* (based on *cox1*, *cox2*, *rrnS* and *rrnL* nucleotide sequences) forming a lineage with *B*. (*Gymnodacus*) *calophylli* instead of with the subgenus *Bactrocera* [43]. It is evident that a broader taxon sampling and the use of mitogenomes will enable a better understanding of the phylogeny of *Bactrocera* and other tephritid fruit flies.

In summary, we have successfully sequenced the whole mitochondrial genomes of *B*. *latifrons*, *B*. *melastomatos* and *B*. *umbrosa* by using next generation sequencing technologies. The mitochondrial genome features are similar to other tephritid fruit flies. The phylogenetic species tree based on 13 PCGs is in general concordant with that based on 15 mt-genes. Based on concatenated 13 protein-coding genes and 15 mt-genes of the mitogenome, *B*. *latifrons* and *B*. *umbrosa* form a sister lineage most basal to the subgenus *Bactrocera*. The subgenus *Bactrocera* is monophyletic. The whole mitogenomes will serve as a useful dataset for studying the genetics, systematics and phylogenetic relationships of the many species of *Bactrocera* genus in particular, and tephritid fruit flies in general.

## Supporting Information

S1 Fig Cloverleaf structure of the 22 inferred tRNAs in the mitogenome of *Bactrocera latifrons*. The cloverleaf structure for *trnF* lacked the TψC-loop, and *trnS1* lacked the DHU-loop.
(DOCX)

S2 Fig Cloverleaf structure of the 22 inferred tRNAs in the mitogenome of *Bactrocera melastomatos*. The cloverleaf structure for *trnF* lacked the TψC-loop, and *trnS1* lacked the DHU-loop.
(DOCX)

S3 Fig Cloverleaf structure of the 22 inferred tRNAs in the mitogenome of *Bactrocera umbrosa*. The cloverleaf structure for *trnC* and *trnF* lacked the TψC-loop, and *trnS1* lacked the DHU-stem.
(DOCX)

S1 Table Characteristics of the mitochondrial genome of *Bactrocera latifrons*. The anticodon of each tRNAs is shown in bracket. J (+) or N (-) indicates gene directions.
(DOCX)

S2 Table Characteristics of the mitochondrial genome of *Bactrocera melastomatos*. The anticodon of each tRNAs is shown in bracket. J (+) or N (-) indicates gene directions.
(DOCX)

S3 Table Characteristics of the mitochondrial genome of *Bactrocera umbrosa*. The anticodon of each tRNAs is shown in bracket. J (+) or N (-) indicates gene directions.
(DOCX)

S4 Table Start/stop codon of protein-coding genes (PCGs) of *Bactrocera* taxa of the subgenus *Bactrocera*. Highlighted text indicates difference in start/stop codon with reference to *B*. *latifrons*.
(DOCX)

S5 Table Nucleotide composition of whole mitogenome, protein-coding genes, rRNA genes and control region of *Bactrocera latifrons*. (DOCX)

S6 Table Nucleotide composition of whole mitogenome, protein-coding genes, rRNA genes and control region of *Bactrocera melastomatos*. (DOCX)

S7 Table Nucleotide composition of whole mitogenome, protein-coding genes, rRNA genes and control region of *Bactrocera umbrosa*. (DOCX)
