# Supplementary material for: Complete Mitochondrial Genome of Three Bactrocera Fruit Flies of Subgenus Bactrocera (Diptera: Tephritidae) and Their Phylogenetic Implications
Source: PLoS One. 2016 Feb 3;11(2):e0148201. doi: 10.1371/journal.pone.0148201 (PMC4739531; doi:10.1371/journal.pone.0148201)
Supplement: S3 Fig — The cloverleaf structure for trnC and trnF lacked the TψC-loop, and trnS1 lacked the DHU-stem. (DOCX) [file pone.0148201.s003.docx]

**
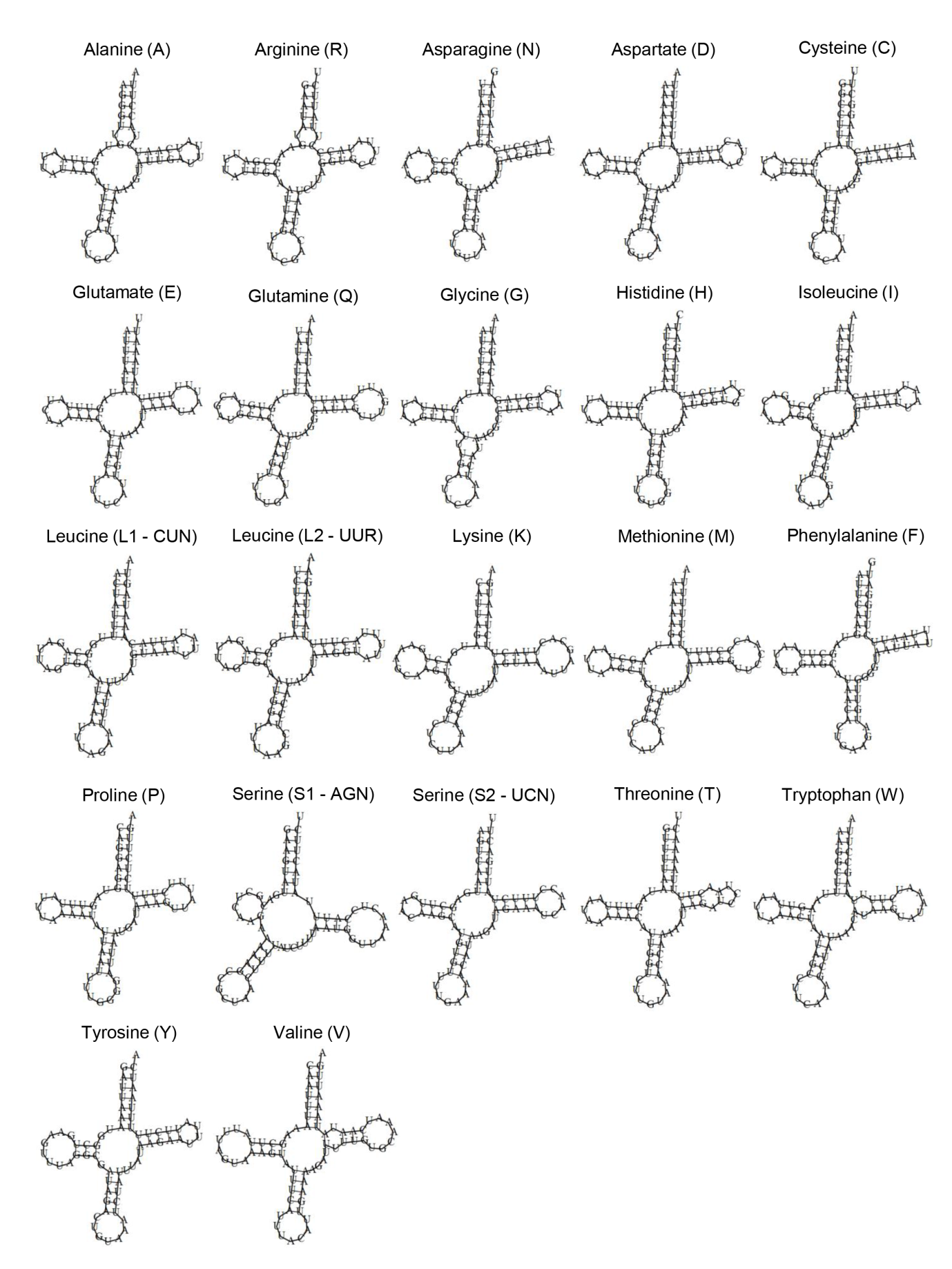
**

**S3 Fig. Cloverleaf structure of the 22 inferred tRNAs in the mitogenome of *Bactrocera umbrosa*.** The cloverleaf structure for *trnC* and *trnF* lacked the TψC-loop, and *trnS1* lacked the DHU-stem.
