# Supplementary material for: Complete Mitochondrial Genome of Three Bactrocera Fruit Flies of Subgenus Bactrocera (Diptera: Tephritidae) and Their Phylogenetic Implications
Source: PLoS One. 2016 Feb 3;11(2):e0148201. doi: 10.1371/journal.pone.0148201 (PMC4739531; doi:10.1371/journal.pone.0148201)
Supplement: S1 Table — The anticodon of each tRNAs is shown in bracket. J (+) or N (-) indicates gene directions. (DOCX) [file pone.0148201.s004.docx]

**S1 Table. Characteristics of the mitochondrial genome of *Bactrocera latifrons.*** The anticodon of each tRNAs is shown in bracket. J (+) or N (-) indicates gene directions.

| Gene | Location | Strand | Size (bp) | Intergenic sequence | Start/stop codon |
| --- | --- | --- | --- | --- | --- |
| *trnI*(gat) | 1 – 66 | J | 66 | -3 |  |
| *trnQ*(ttg) | 64 – 132 | N | 69 | 94 |  |
| *trnM*(cat) | 227 – 295 | J | 69 |  |  |
| *nad2* | 296 – 1318 | J | 1023 | 13 | ATT/TAA |
| *trnW*(tca) | 1332 – 1400 | J | 69 | -8 |  |
| *trnC*(gca) | 1393 – 1455 | N | 63 | 39 |  |
| *trnY*(gta) | 1495 – 1561 | N | 67 | -2 |  |
| *cox1* | 1560 – 3094 | J | 1535 |  | TCG/TA |
| *trnL2*(taa) | 3095 – 3160 | J | 66 | 4 |  |
| *cox2* | 3165 – 3854 | J | 690 | 4 | ATG/TAA |
| *trnK*(ctt) | 3859 – 3929 | J | 71 | 2 |  |
| *trnD*(gtc) | 3932 – 3999 | J | 68 |  |  |
| *atp8* | 4000 – 4161 | J | 162 | -7 | GTG/TAA |
| *atp6* | 4155 – 4832 | J | 678 | -1 | ATG/TAA |
| *cox3* | 4832 – 5620 | J | 789 | 9 | ATG/TAA |
| *trnG*(tcc) | 5630 – 5694 | J | 65 |  |  |
| *nad3* | 5695 – 6046 | J | 352 |  | ATT/T |
| *trnA*(tgc) | 6047 – 6111 | J | 65 | 13 |  |
| *trnR*(tcg) | 6125 – 6188 | J | 64 | 43 |  |
| *trnN*(gtt) | 6232 – 6296 | J | 65 |  |  |
| *trnS1*(gct) | 6297 – 6364 | J | 68 |  |  |
| *trnE*(ttc) | 6365 – 6429 | J | 65 | 18 |  |
| *trnF*(gaa) | 6448 – 6512 | N | 65 |  |  |
| *nad5* | 6513 – 8229 | N | 1717 | 15 | ATT/T |
| *trnH*(gtg) | 8245 – 8309 | N | 65 |  |  |
| *nad4* | 8310 – 9650 | N | 1341 | -7 | ATG/TAG |
| *nad4l* | 9644 – 9940 | N | 297 | 2 | ATG/TAA |
| *trnT*(tgt) | 9943 – 10007 | J | 65 |  |  |
| *trnP*(tgg) | 10008 – 10073 | N | 66 | 2 |  |
| *nad6* | 10076 – 10600 | J | 525 | -1 | ATT/TAA |
| *cob* | 10600 – 11734 | J | 1135 |  | ATG/T |
| *trnS2*(tga) | 11735 – 11802 | J | 68 | 15 |  |
| *nad1* | 11818 – 12757 | N | 940 | 10 | ATA/T |
| *trnL1*(tag) | 12768 – 12832 | N | 65 |  |  |
| *rrnL* | 12833 – 14164 | N | 1332 |  |  |
| *trnV(*tac) | 14165 – 14236 | N | 72 |  |  |
| *rrnS* | 14237 – 15024 | N | 788 |  |  |
| Control region | 15025 – 15977 | J | 953 |  |  |
