# Supplementary material for: Complete Mitochondrial Genome of Three Bactrocera Fruit Flies of Subgenus Bactrocera (Diptera: Tephritidae) and Their Phylogenetic Implications
Source: PLoS One. 2016 Feb 3;11(2):e0148201. doi: 10.1371/journal.pone.0148201 (PMC4739531; doi:10.1371/journal.pone.0148201)
Supplement: S2 Table — The anticodon of each tRNAs is shown in bracket. J (+) or N (-) indicates gene directions. (DOCX) [file pone.0148201.s005.docx]

**S2 Table. Characteristics of the mitochondrial genome of *Bactrocera melastomatos.*** The anticodon of each tRNAs is shown in bracket. J (+) or N (-) indicates gene directions.

| Gene | Location | Strand | Size (bp) | Intergenic sequence | Start/stop codon |
| --- | --- | --- | --- | --- | --- |
| *trnI*(gat) | 1 – 66 | J | 66 | -3 |  |
| *trnQ*(ttg) | 64 – 132 | N | 69 | 82 |  |
| *trnM*(cat) | 215 – 283 | J | 69 |  |  |
| *nad2* | 284 – 1306 | J | 1023 | 12 | ATT/TAA |
| *trnW*(tca) | 1319 – 1387 | J | 69 | -8 |  |
| *trnC*(gca) | 1380 – 1443 | N | 64 | 39 |  |
| *trnY*(gta) | 1483 – 1549 | N | 67 | -2 |  |
| *cox1* | 1548 – 3082 | J | 1535 |  | TCG/TA |
| *trnL2*(taa) | 3083 – 3148 | J | 66 | 4 |  |
| *cox2* | 3153 – 3842 | J | 690 | 4 | ATG/TAA |
| *trnK*(ctt) | 3847 – 3917 | J | 71 | 2 |  |
| *trnD*(gtc) | 3920 – 3986 | J | 67 |  |  |
| *atp8* | 3987 – 4148 | J | 162 | -7 | GTG/TAA |
| *atp6* | 4142 – 4819 | J | 678 | -1 | ATG/TAA |
| *cox3* | 4819 – 5607 | J | 789 | 9 | ATG/TAA |
| *trnG*(tcc) | 5617 – 5681 | J | 65 |  |  |
| *nad3* | 5682 – 6033 | J | 352 |  | ATC/T |
| *trnA*(tgc) | 6034 – 6098 | J | 65 | 12 |  |
| *trnR*(tcg) | 6111 – 6174 | J | 64 | 35 |  |
| *trnN*(gtt) | 6210 – 6274 | J | 65 |  |  |
| *trnS1*(gct) | 6275 – 6342 | J | 68 |  |  |
| *trnE*(ttc) | 6343 – 6409 | J | 67 | 18 |  |
| *trnF*(gaa) | 6428 – 6492 | N | 65 |  |  |
| *nad5* | 6493 – 8212 | N | 1720 | 15 | ATT/T |
| *trnH*(gtg) | 8228 – 8293 | N | 66 |  |  |
| *nad4* | 8294 – 9634 | N | 1341 | -7 | ATG/TAG |
| *nad4l* | 9628 – 9924 | N | 297 | 2 | ATG/TAA |
| *trnT*(tgt) | 9927 – 9991 | J | 65 |  |  |
| *trnP*(tgg) | 9992 – 10057 | N | 66 | 2 |  |
| *nad6* | 10060 – 10584 | J | 525 | -1 | ATC/TAA |
| *cob* | 10584 – 11718 | J | 1135 |  | ATG/T |
| *trnS2*(tga) | 11719 – 11785 | J | 67 | 15 |  |
| *nad1* | 11801 – 12740 | N | 940 | 10 | ATA/T |
| *trnL1*(tag) | 12751 – 12815 | N | 65 |  |  |
| *rrnL* | 12816 – 14142 | N | 1327 |  |  |
| *trnV(*tac) | 14143 – 14214 | N | 72 |  |  |
| *rrnS* | 14215 – 15001 | N | 787 |  |  |
| Control region | 15002 – 15954 | J | 953 |  |  |
