# Supplementary material for: Complete Mitochondrial Genome of Three Bactrocera Fruit Flies of Subgenus Bactrocera (Diptera: Tephritidae) and Their Phylogenetic Implications
Source: PLoS One. 2016 Feb 3;11(2):e0148201. doi: 10.1371/journal.pone.0148201 (PMC4739531; doi:10.1371/journal.pone.0148201)
Supplement: S3 Table — The anticodon of each tRNAs is shown in bracket. J (+) or N (-) indicates gene directions. (DOCX) [file pone.0148201.s006.docx]

**S3 Table. Characteristics of the mitochondrial genome of *Bactrocera umbrosa.*** The anticodon of each tRNAs is shown in bracket. J (+) or N (-) indicates gene directions.

| Gene | Location | Strand | Size (bp) | Intergenic Sequence | Start/stop codon |
| --- | --- | --- | --- | --- | --- |
| *trnI*(gat) | 1 – 66 | J | 66 | -3 |  |
| *trnQ*(ttg) | 64 – 132 | N | 69 | 79 |  |
| *trnM*(cat) | 212 – 280 | J | 69 |  |  |
| *nad2* | 281 – 1303 | J | 1023 | 10 | ATT/TAA |
| *trnW*(tca) | 1314 – 1382 | J | 69 | -8 |  |
| *trnC*(gca) | 1375 – 1437 | N | 63 | 15 |  |
| *trnY*(gta) | 1453 – 1519 | N | 67 | -2 |  |
| *cox1* | 1518 – 3052 | J | 1535 |  | TCG/TA |
| *trnL2*(taa) | 3053 – 3118 | J | 66 | 4 |  |
| *cox2* | 3123 – 3812 | J | 690 | 4 | ATG/TAA |
| *trnK*(ctt) | 3817 – 3887 | J | 71 | 3 |  |
| *trnD*(gtc) | 3891 – 3957 | J | 67 |  |  |
| *atp8* | 3958 – 4119 | J | 162 | -7 | ATG/TAA |
| *atp6* | 4113 – 4790 | J | 678 | -1 | ATG/TAA |
| *cox3* | 4790 – 5578 | J | 789 | 9 | ATG/TAA |
| *trnG*(tcc) | 5588 – 5652 | J | 65 |  |  |
| *nad3* | 5653 – 6004 | J | 352 |  | ATT/T |
| *trnA*(tgc) | 6005 – 6069 | J | 65 | 12 |  |
| *trnR*(tcg) | 6082 – 6145 | J | 64 | 13 |  |
| *trnN*(gtt) | 6159 – 6223 | J | 65 |  |  |
| *trnS1*(gct) | 6224 – 6291 | J | 68 |  |  |
| *trnE*(ttc) | 6292 – 6357 | J | 66 | 18 |  |
| *trnF*(gaa) | 6376 – 6440 | N | 65 |  |  |
| *nad5* | 6441 – 8160 | N | 1720 | 15 | ATT/T |
| *trnH*(gtg) | 8176 – 8240 | N | 65 |  |  |
| *nad4* | 8241 – 9581 | N | 1341 | -7 | ATG/TAG |
| *nad4l* | 9575 – 9871 | N | 297 | 2 | ATG/TAA |
| *trnT*(tgt) | 9874 – 9938 | J | 65 |  |  |
| *trnP*(tgg) | 9939 – 10004 | N | 66 | 2 |  |
| *nad6* | 10007 – 10531 | J | 525 | -1 | ATT/TAA |
| *cob* | 10531 – 11665 | J | 1135 |  | ATG/T |
| *trnS2*(tga) | 11666 – 11732 | J | 67 | 15 |  |
| *nad1* | 11748 – 12687 | N | 940 | 10 | ATA/T |
| *trnL1*(tag) | 12698 – 12762 | N | 65 |  |  |
| *rrnL* | 12763 – 14093 | N | 1331 |  |  |
| *trnV(*tac) | 14094 – 14165 | N | 72 |  |  |
| *rrnS* | 14166 – 14954 | N | 789 |  |  |
| Control region | 14955 – 15898 | J | 944 |  |  |
