# Supplementary material for: Complete Mitochondrial Genome of Three Bactrocera Fruit Flies of Subgenus Bactrocera (Diptera: Tephritidae) and Their Phylogenetic Implications
Source: PLoS One. 2016 Feb 3;11(2):e0148201. doi: 10.1371/journal.pone.0148201 (PMC4739531; doi:10.1371/journal.pone.0148201)
Supplement: S4 Table — Highlighted text indicates difference in start/stop codon with reference to B. latifrons. (DOCX) [file pone.0148201.s007.docx]

**S4 Table. Start/stop codon of protein-coding genes (PCGs) of *Bactrocera* taxa of the subgenus *Bactrocera*.** Highlighted text indicates difference in start/stop codon with reference to *B. latifrons*.

| Gene | *B.*  *latifrons* | *B.*  *melastomatos* | *B.*  *umbrosa* | *B.*  *arecae* | *B.*  *correcta* | *B.*  *dorsalis* | *B.*  *carambolae* | *B.*  *tryoni* | *B.*  *zonata* |
| --- | --- | --- | --- | --- | --- | --- | --- | --- | --- |
| *nad2* | ATT/TAA | ATT/TAA | ATT/TAA | ATT/TAA | ATT/TAA | ATT/TAA | ATT/TAA | ATT/TAA | ATT/TAA |
| *cox1* | TCG/TA | TCG/TA | TCG/TA | TCG/TA | TCG/TA | TCG/TA | TCG/TA | TCG/TA | TCG/TA |
| *cox2* | ATG/TAA | ATG/TAA | ATG/TAA | ATG/TAA | ATG/TAA | ATG/TAA | ATG/TAA | ATG/TAA | ATG/TAA |
| *atp8* | GTG/TAA | GTG/TAA | ATG/TAA | GTG/TAA | GTG/TAA | GTG/TAA | GTG/TAA | GTG/TAA | GTG/TAA |
| *atp6* | ATG/TAA | ATG/TAA | ATG/TAA | ATG/TAA | ATG/TAA | ATG/TA | ATG/TAA | ATG/TAA | ATG/TAA |
| *cox3* | ATG/TAA | ATG/TAA | ATG/TAA | ATG/TAA | ATG/TAA | ATG/TAA | ATG/TAA | ATG/TAA | ATG/TAA |
| *nad3* | ATT/T | ATC/T | ATT/T | ATT/T | ATT/TAG | ATT/T | ATT/T | ATT/T | ATT/T |
| *nad5* | ATT/T | ATT/T | ATT/T | ATC/TAA | ATT/TA | ATT/T | ATT/TA | ATT/T | ATT/TA |
| *nad4* | ATG/TAG | ATG/TAG | ATG/TAG | ATG/TAG | ATG/TAG | ATG/TAG | ATG/TAG | ATG/TAG | ATG/TAG |
| *nad4l* | ATG/TAA | ATG/TAA | ATG/TAA | ATG/TAA | ATG/TAA | ATG/TAA | ATG/TAA | ATG/TAA | ATG/TAA |
| *nad6* | ATT/TAA | ATC/TAA | ATT/TAA | ATT/TAA | ATT/TAA | ATT/TA | ATT/TAA | ATG/TAA | ATT/TAA |
| *cob* | ATG/T | ATG/T | ATG/T | ATG/TAG | ATG/TAG | ATG/T | ATG/TAG | ATG/T | ATG/TAG |
| *nad1* | ATA/T | ATA/T | ATA/T | ATA/T | ATA/T | ATA/T | ATA/T | ATA/T | ATA/T |
