# Supplementary material for: Complete Mitochondrial Genome of Three Bactrocera Fruit Flies of Subgenus Bactrocera (Diptera: Tephritidae) and Their Phylogenetic Implications
Source: PLoS One. 2016 Feb 3;11(2):e0148201. doi: 10.1371/journal.pone.0148201 (PMC4739531; doi:10.1371/journal.pone.0148201)
Supplement: S5 Table — (DOCX) [file pone.0148201.s008.docx]

**S5 Table. Nucleotide composition of whole mitogenome, protein-coding genes, rRNA genes and control region of *Bactrocera latifrons*.**

| Region | A% | C% | G% | T% | A+T% | G+C% | AT skew | GC skew |
| --- | --- | --- | --- | --- | --- | --- | --- | --- |
| Whole mitogenome | 38.7 | 18.3 | 10.6 | 32.4 | 71.1 | 28.9 | 0.089 | -0.266 |
| *nad2* | 34.0 | 19.0 | 10.1 | 36.9 | 70.9 | 29.1 | -0.041 | -0.306 |
| *cox1* | 29.7 | 22.0 | 16.2 | 32.1 | 61.8 | 38.2 | -0.039 | -0.152 |
| *cox2* | 31.6 | 21.6 | 14.8 | 32.0 | 63.6 | 36.4 | -0.006 | -0.187 |
| *atp8* | 35.2 | 17.9 | 9.3 | 37.7 | 72.8 | 27.2 | -0.034 | -0.316 |
| *atp6* | 29.7 | 22.6 | 12.2 | 35.5 | 65.2 | 34.8 | -0.089 | -0.299 |
| *cox3* | 29.8 | 22.4 | 14.8 | 33.0 | 62.7 | 37.3 | -0.051 | -0.204 |
| *nad3* | 31.8 | 19.6 | 10.2 | 38.4 | 70.2 | 29.8 | -0.094 | -0.315 |
| *nad5* | 45.8 | 19.2 | 9.5 | 25.5 | 71.3 | 28.7 | 0.285 | -0.338 |
| *nad4* | 47.1 | 19.1 | 9.5 | 24.3 | 71.4 | 28.6 | 0.319 | -0.336 |
| *nad4l* | 49.2 | 15.8 | 7.7 | 27.3 | 76.4 | 23.6 | 0.287 | -0.343 |
| *nad6* | 36.8 | 19.6 | 7.0 | 36.6 | 73.3 | 26.7 | 0.003 | -0.472 |
| *cob* | 31.3 | 22.5 | 12.9 | 33.3 | 64.6 | 35.4 | -0.031 | -0.271 |
| *nad1* | 47.7 | 18.7 | 9.6 | 24.0 | 71.7 | 28.3 | 0.331 | -0.322 |
| *rrnS* | 40.6 | 16.6 | 9.0 | 33.8 | 74.4 | 25.6 | 0.091 | -0.297 |
| *rrnL* | 42.6 | 14.0 | 7.1 | 36.3 | 78.9 | 21.1 | 0.080 | -0.327 |
| Control region | 46.4 | 8.2 | 5.0 | 40.4 | 86.8 | 13.2 | 0.069 | -0.242 |
