# Supplementary material for: Complete Mitochondrial Genome of Three Bactrocera Fruit Flies of Subgenus Bactrocera (Diptera: Tephritidae) and Their Phylogenetic Implications
Source: PLoS One. 2016 Feb 3;11(2):e0148201. doi: 10.1371/journal.pone.0148201 (PMC4739531; doi:10.1371/journal.pone.0148201)
Supplement: S6 Table — (DOCX) [file pone.0148201.s009.docx]

**S6 Table. Nucleotide composition of whole mitogenome, protein-coding genes, rRNA genes and control region of *Bactrocera melastomatos*.**

| Region | A% | C% | G% | T% | A+T% | G+C% | AT skew | GC skew |
| --- | --- | --- | --- | --- | --- | --- | --- | --- |
| Whole mitogenome | 39.6 | 16.4 | 9.8 | 34.2 | 73.8 | 26.2 | 0.073 | -0.252 |
| *nad2* | 35.1 | 15.8 | 8.5 | 40.6 | 75.7 | 24.3 | -0.073 | -0.300 |
| *cox1* | 30.6 | 19.5 | 15.6 | 34.3 | 65.0 | 35.0 | -0.057 | -0.111 |
| *cox2* | 32.7 | 20.3 | 13.5 | 33.5 | 66.2 | 33.8 | -0.012 | -0.201 |
| *atp8* | 36.4 | 16.7 | 8.6 | 38.3 | 74.7 | 25.3 | -0.025 | -0.320 |
| *atp6* | 29.9 | 21.7 | 11.4 | 37.0 | 67.0 | 33.0 | -0.106 | -0.312 |
| *cox3* | 30.8 | 18.6 | 14.1 | 36.5 | 67.3 | 32.7 | -0.085 | -0.138 |
| *nad3* | 33.0 | 15.9 | 9.1 | 42.0 | 75.0 | 25.0 | -0.120 | -0.272 |
| *nad5* | 46.0 | 17.0 | 9.0 | 28.0 | 74.0 | 26.0 | 0.243 | -0.308 |
| *nad4* | 48.8 | 17.0 | 8.2 | 26.0 | 74.8 | 25.2 | 0.305 | -0.349 |
| *nad4l* | 49.5 | 16.2 | 8.4 | 25.9 | 75.4 | 24.6 | 0.313 | -0.317 |
| *nad6* | 38.5 | 15.4 | 5.9 | 40.2 | 78.7 | 21.3 | -0.022 | -0.446 |
| *cob* | 32.0 | 19.6 | 12.5 | 35.9 | 67.8 | 32.2 | -0.058 | -0.220 |
| *nad1* | 48.8 | 17.9 | 8.9 | 24.4 | 73.2 | 26.8 | 0.333 | -0.336 |
| *rrnS* | 41.2 | 16.3 | 9.1 | 33.4 | 74.6 | 25.4 | 0.105 | -0.283 |
| *rrnL* | 43.2 | 13.1 | 6.7 | 37.0 | 80.2 | 19.8 | 0.077 | -0.323 |
| Control region | 47.1 | 6.9 | 4.1 | 41.9 | 89.0 | 11.0 | 0.058 | -0.254 |
