# Supplementary material for: Complete Mitochondrial Genome of Three Bactrocera Fruit Flies of Subgenus Bactrocera (Diptera: Tephritidae) and Their Phylogenetic Implications
Source: PLoS One. 2016 Feb 3;11(2):e0148201. doi: 10.1371/journal.pone.0148201 (PMC4739531; doi:10.1371/journal.pone.0148201)
Supplement: S7 Table — (DOCX) [file pone.0148201.s010.docx]

**S7 Table. Nucleotide composition of whole mitogenome, protein-coding genes, rRNA genes and control region of *Bactrocera umbrosa*.**

| Region | A% | C% | G% | T% | A+T% | G+C% | AT skew | GC skew |
| --- | --- | --- | --- | --- | --- | --- | --- | --- |
| Whole mitogenome | 38.2 | 18.3 | 11.2 | 32.3 | 70.5 | 29.5 | 0.084 | -0.241 |
| *nad2* | 32.2 | 19.5 | 10.7 | 37.6 | 69.8 | 30.2 | -0.077 | -0.291 |
| *cox1* | 28.1 | 21.7 | 17.8 | 32.4 | 60.5 | 39.5 | -0.071 | -0.099 |
| *cox2* | 32.9 | 22.2 | 14.8 | 30.1 | 63.0 | 37.0 | 0.044 | -0.200 |
| *atp8* | 34.6 | 24.7 | 8.6 | 32.1 | 66.7 | 33.3 | 0.037 | -0.483 |
| *atp6* | 29.6 | 23.5 | 11.8 | 35.1 | 64.7 | 35.3 | -0.085 | -0.331 |
| *cox3* | 29.0 | 21.0 | 15.7 | 34.2 | 63.2 | 36.8 | -0.082 | -0.144 |
| *nad3* | 31.8 | 18.7 | 11.4 | 38.1 | 69.9 | 30.1 | -0.090 | -0.243 |
| *nad5* | 45.5 | 18.5 | 10.4 | 25.6 | 71.0 | 29.0 | 0.280 | -0.279 |
| *nad4* | 46.5 | 18.6 | 9.9 | 25.0 | 71.4 | 28.6 | 0.301 | -0.304 |
| *nad4l* | 49.2 | 16.5 | 9.1 | 25.2 | 74.4 | 25.6 | 0.323 | -0.289 |
| *nad6* | 34.1 | 19.4 | 8.8 | 37.7 | 71.8 | 28.2 | -0.050 | -0.376 |
| *cob* | 30.2 | 22.1 | 14.0 | 33.7 | 63.9 | 36.1 | -0.055 | -0.224 |
| *nad1* | 47.4 | 20.2 | 10.1 | 22.3 | 69.7 | 30.3 | 0.360 | -0.333 |
| *rrnS* | 40.3 | 16.9 | 9.5 | 33.3 | 73.6 | 26.4 | 0.095 | -0.280 |
| *rrnL* | 43.2 | 14.6 | 6.4 | 35.8 | 79.0 | 21.0 | 0.094 | -0.390 |
| Control region | 45.2 | 7.2 | 6.6 | 41.0 | 86.2 | 13.8 | 0.049 | -0.043 |
